# Selective Targeting of Breast Cancer by Tafuramycin A Using SMA-Nanoassemblies

**DOI:** 10.3390/molecules26123532

**Published:** 2021-06-09

**Authors:** Ibrahim M. El-Deeb, Valeria Pittala, Diab Eltayeb, Khaled Greish

**Affiliations:** 1 Department of Medical Sciences, Royal College of Surgeons in Ireland, Medical University of Bahrain, Busaiteen 228, Bahrain; imeldeeb@gmail.com; 2 Department of Drug and Health Science, University of Catania, 95125 Catania, Italy; 3 Department of Molecular Medicine, Arabian Gulf University, Manama 329, Bahrain; diabad@agu.edu.bh

**Keywords:** duocarmycin, tafuramycin A, TNBC, poly(styrene-co-maleic acid) micelles, nanoformulation, EPR effect, nanomedicine

## Abstract

Triple-negative breast cancer (TNBC) is a heterogeneous subtype of tumors that tests negative for estrogen receptors, progesterone receptors, and excess HER2 protein. The mainstay of treatment remains chemotherapy, but the therapeutic outcome remains inadequate. This paper investigates the potential of a duocarmycin derivative, tafuramycin A (TFA), as a new and more effective chemotherapy agent in TNBC treatment. To this extent, we optimized the chemical synthesis of TFA, and we encapsulated TFA in a micellar system to reduce side effects and increase tumor accumulation. In vitro and in vivo studies suggest that both TFA and SMA–TFA possess high anticancer effects in TNBC models. Finally, the encapsulation of TFA offered a preferential avenue to tumor accumulation by increasing its concentration at the tumor tissues by around four times in comparison with the free drug. Overall, the results provide a new potential strategy useful for TNBC treatment.

## 1. Introduction

The International Agency for Research on Cancer recently reported for the year 2020 an incidence of almost 20 million new cancer cases and around 10 million cancer deaths [1]. Breast tumor has exceeded lung cancer as the most frequently diagnosed cancer in females, with a reported incidence of 2.3 million new cases. Progress in the treatment of breast cancer has been reported in recent decades, especially with the identification of molecular markers for targeted therapies. Well-established breast cancer molecular markers with prognostic and/or therapeutic significance include estrogen receptor (ER), progesterone receptor (PR), and human epidermal growth factor receptor 2 (HER2) [2]. Triple-negative breast cancer (TNBC) is defined by the deficiency of the aforementioned therapeutic markers and represents a variegated subfamily of tumors showing an aggressive clinical profile manifested by rapid proliferation, high recurrence and chemoresistance risk, fast progression, distant metastasis, and poor prognosis [3]. TNBC is a major clinical challenge with only limited efficacious treatment modalities. Available options are surgery, radio-, immuno-, and photothermal therapy [4,5,6]. However, in addition to the aforementioned treatments, the primary therapy option for TNBC treatment is chemotherapy and still represents the mainstay [6]. However, given poor outcomes, the frequent occurrence of relapses and chemoresistance, and the lack of effective cures, there is an immense effort in finding a new treatment to treat TNBC. New approaches include the use of nanomedicine to deliver drug combinations selectively at the tumor site, targeting the acidic microenvironment, a hyaluronic acid (HA) coating on the nanoparticles surface to target CD44-overexpressed tumor cells, the use of natural substances in combination with classical chemotherapy, and others [7,8]. 

Duocarmycins are a family of alkaloids isolated from *Streptomyces* sp. [9]. Following their discovery, these natural derivatives have long fascinated researchers due to their notably anticancer activity, exclusive mechanism of action, and efficacy in multidrug-resistant tumor models. These molecules exert their cytotoxic effect through binding at the minor groove of AT-rich sequences in the DNA and by covalently alkylating adenine-N3 [10]. Intense structure–activity relationship studies (SARs) have been performed, achieving great improvements in the pharmacological profile, and a number of formulations have been investigated [11]. However, duocarmycin analogs failed to reach the market as anticancer drugs because of their severe toxicity, particularly to bone marrow and liver [12,13,14,15]. Nevertheless, this class of compounds is still receiving great interest from the scientific community due to the inherent ability of duocarmycins to escape classical resistance phenomena and to hold attractive activity in multidrug-resistant cells. Accordingly, considerable efforts from both academia and industry are still being made to optimize duocarmycin derivatives, with a special focus on increasing the therapeutic index for patient advantage [16]. Strategies to improve the safety of duocarmycin derivatives are focusing on the development of prodrugs activated at the site of action, antibody–drug conjugates, small-molecule drug conjugates, peptide–drug conjugates, and chemical modification/simplification of the duocarmicin’s structure [16]. In addition, another convenient avenue commonly explored to reduce side effects is the nanoformulation of active compounds. The incorporation of a bioactive compound in a micellar structure may reduce metabolization, limit side effects, and improve tumor accumulation through the enhanced permeability and retention effect (EPR) [17,18].

**Figure 1:**
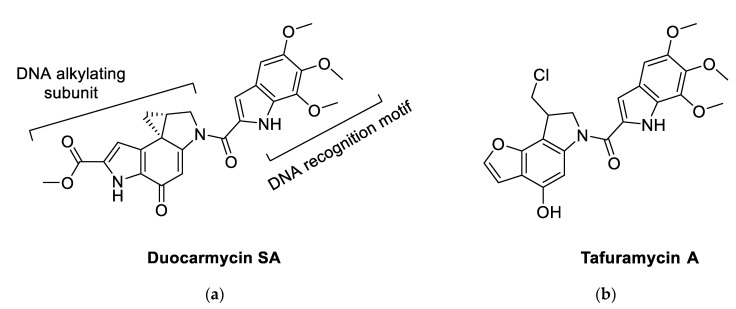
Chemical structure of (**a**) duocarmycin SA and (**b**) tafuramycin A.

From a chemical point of view, duocarmycin derivatives, bearing a cyclopropapyrroloindole nucleus as (+)-duocarmycin SA (DSA, Figure 1), consist of two different portions: a DNA-recognition motif (DNA-RM) and a pharmacophore in control of DNA alkylation. SARs around the DNA-alkylating subunit dramatically affect the anticancer potency in the subnanomolar range, with compounds inducing cell apoptosis via S-phase inhibition and following cell cycle arrest [19,20]. A number of these SARs were successful in simplifying the structure of duocarmycins while maintaining high potency. An example of these simplified duocarmycin derivatives is represented by tafuramycin A (TFA, Figure 1) [21]. TFA is a potent anticancer and parasite-attenuating agent that has been recently used for the attenuation of plasmodium malaria parasites for the production of malaria vaccine [22]. TFA, is a *seco*-prodrug that dehydrochlorinates inside the cell to originate the active cyclopropane-containing molecule that later alkylates DNA. When tested against a panel of tumoral cell lines, TFA displayed activity in the nanomolar range against selected solid tumors and, at the same time, showed only mild toxicity against murine bone marrow cells [21].

With the aim of discovering ways to unlock the immense potential of duocarmycin derivatives as new and more effective chemotherapies in TNBC, TFA was loaded on styrene–maleic acid (SMA) micelles to increase the tumor concentration of the active principle through the EPR effect and to prevent off-target effects. Obtained compounds and SMA–tafuramycin A (SMA–TFA) were tested in MDA-MB-231, MDA-MB-468, 4T1, and MCF7 breast cancer cell lines and in vivo in a syngeneic model of TNBC. The cell lines chosen for the present study embody a spectrum of normally used breast cancer cell lines of both hormonal responsiveness and TNBC of human and murine derivation. The use of these cell lines will allow the comparison of TFA formulation in different biological settings and, additionally, enable the comparison of our results to others research in the field.

## 2. Results

### 2.1. Chemistry

**Scheme 1:**
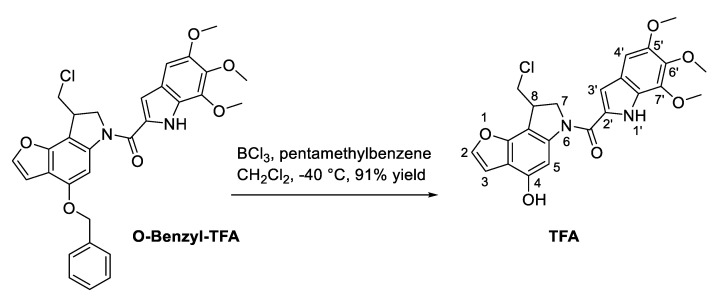
Optimized synthesis of TFA by the benzyl ether deprotection of *O*-benzyl–TFA.

An optimized, high-yielding, and more efficient synthesis of TFA has been previously reported by our research group [23]. In this optimized synthesis, the protecting *O*-benzyl group was replaced by a *p-*methoxybenzyl moiety to allow for an alternative deprotection method (acid deprotection instead of catalytic dehydrogenation) in order to avoid partial reduction of the furan ring double bond. While it proved efficient and high yielding, trials using this method for the scale-up synthesis of gram quantities of TFA were associated with a drop in the overall yield. Accordingly, a new deprotection method for the *O-*benzyl-protecting group in *O*-benzyl–TFA was employed (Scheme 1). *O*-benzyl–TFA was synthesized in good overall yields following our reported methods [23]. The *O-*benzyl ether was removed under the effect of borontrichloride in the presence of petamethylbenzene in DCM at −40 °C to yield TFA exclusively at 91% yield. The new deprotection step was applicable to gram quantities of the starting material, and the product was obtained at high purity after column chromatography, as clearly demonstrated by ^1^H and ^13^C-NMR spectra (see Supplementary Materials).

### 2.2. SMA–TFA Micelles Synthesis and Characterization

**Figure 2:**
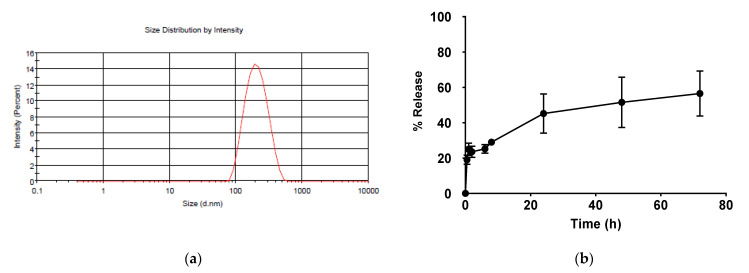
(**a**) Size distribution of SMA–TFA micelles. The size distribution of SMA–TFA micelles was determined using a Malvern Zeta Sizer at a concentration of 1.5 mg/mL in water. Data are expressed as the mean ± SEM (*n* = 3); (**b**) SMA–TFA drug release studies. Cumulative release of TFA from SMA–TFA micelles at pH 7.4 in PBS. Data are presented as the average ± SD (*n* = 3).

SMA–TFA was synthesized and characterized by a low critical micelle concentration (CMC). Furthermore, the structural variation of hydrophobic styrene and hydrophilic maleic groups stimulates the quick construction of SMA micelles and facilitates the encapsulation of TFA. The loading of SMA–TFA was 20%, calculated as the weight ratio of TFA over the total amount of SMA micelle weight. The micelle recovery was calculated as the percentage value between the obtained weight in SMA–TFA divided by the theoretical amount expected. The measurement of average micelle size showed that SMA–TFA micelles were 182 nm with a polydispersity index (PDI) of 0.163, which was determined by dynamic light scattering (DLS) (Figure 2a). As shown in Table 1, the zeta potential of SMA–TFA is negative with a value of –57.7 mV, which can sustain the micelle in the blood circulation for a long time by lowering the clearance of the reticuloendothelial system, allowing accumulation in the tumor.

Thus, the average size of SMA–TFA is within the size range to facilitate its accumulation in tumor tissue by the effect of enhanced permeability and retention (EPR) [24]. Moreover, the release rate of the drug from the micelles was monitored for a period of 72 h (Figure 2b). Within the first 30 min, an abrupt release of ~20% was observed, which might be attributable to TFA association with the SMA shell rather than encapsulation in the micellar core. Followed by a slow-release phase in which it is released, encapsulated TFA with almost half the micelle remains intact for up to 3 days.

### 2.3. Antiproliferative Effect of Free TFA and SMA–TFA Micellar Formulation in Breast Cancer Cells

**Figure 3:**
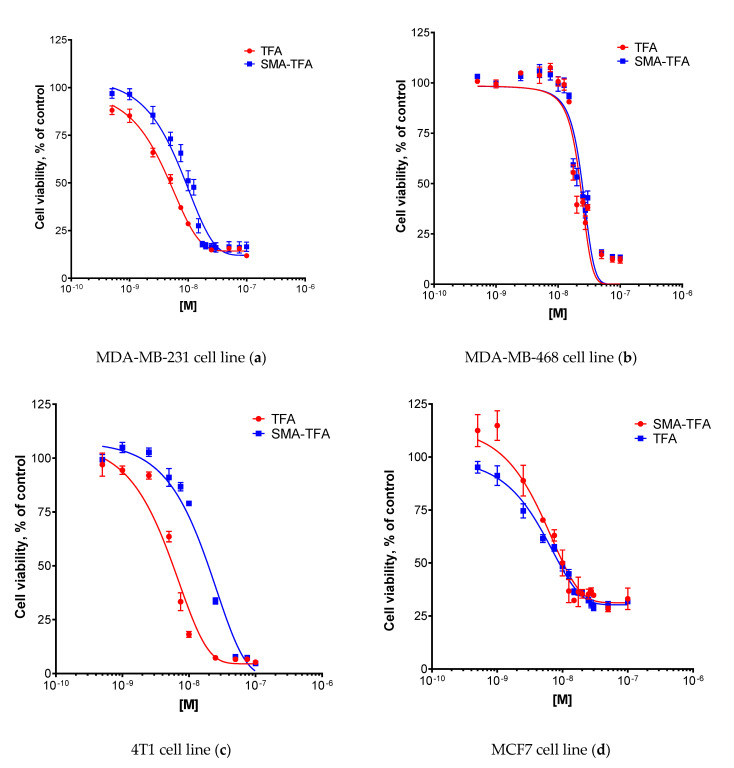
Cytotoxicity of TFA and SMA–TFA (**a**) against MDA-MB-231, (**b**) MDA-MB-468, (**c**) 4T1, and (**d**) MCF7 cells. The cells were treated for 72 h with specific concentrations of TFA and SMA–TFA micelles. The cell number was determined using the SRB assay. Data are expressed as the mean ± SEM (*n* = 3).

The evaluation of the effect of the cellular uptake of SMA–TFA and TFA on cell viability was achieved using four different breast cancer cell lines, namely MDA-MB–231, MDA-MB-468, 4T1, and MCF7 cells (Figure 3). The effect of TFA and SMA–TFA on cell viability was measured using SRB assay [25].

All the treatments with both the TFA free drug and SMA–TFA showed an IC_50_ in the low nanomolar range, spanning from 4.55 to 20.2 nM. The treatment of MDA-MB-468 and MCF7 cells (Figure 3b,d, Table 2) showed comparable cytotoxic activity for the free drug and the micelle formulation (IC_50_ = 18.3 vs. 20.2 nM, and IC_50_ = 4.55 vs. 4.31 nM, respectively), while slight differences between the two preparations were observed in MDA-MB-231 and 4T1 cells (Figure 3a,c, Table 2).

### 2.4. Effect of Free TFA and SMA–TFA Micelles on the Development of 4T1 Tumors

**Figure 4:**
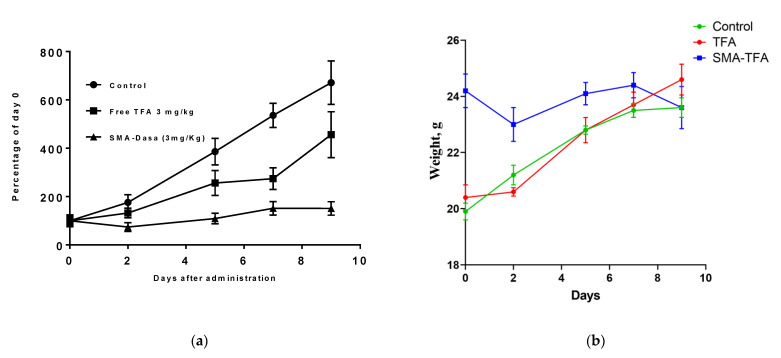
In vivo antitumor activity of TFA and SMA–TFA on 4T1 tumor-bearing Balb/c mice. Mice were treated for 9 days with either TFA (3 mg/kg) and SMA–TFA (3 mg/kg). The control group was injected with PBS (pH 7.4). (**a**) Tumor volume changes and (**b**) body weight changes were monitored over the treatment period. Data are presented as the mean of triplicate experiments ± standard error.

The anticancer activity of TFA and SMA–TFA in a syngeneic model of breast cancer was evaluated using Balb/c mice harboring 4T1 tumors over a treatment period of 9 days (Figure 4a). Figure 4a shows that TFA treatment (3 mg/kg) slightly slowed down tumor growth compared to the control group over the 9-day treatment. Dissimilarly, the treatment with SMA–TFA (3 mg/kg) showed to be much more effective, which seems to have almost entirely stopped the tumor growth for the duration of the study, with a very mild increase between Days 7 and 9. The therapeutic efficacy of TFA treatments was not associated with any statistically significant weight loss during the treatment period, as shown in Figure 4b, while SMA–TFA treatment induced an almost negligible weight loss.

### 2.5. In Vivo Biodistribution of TFA and SMA–TFA Micelles

**Figure 5:**
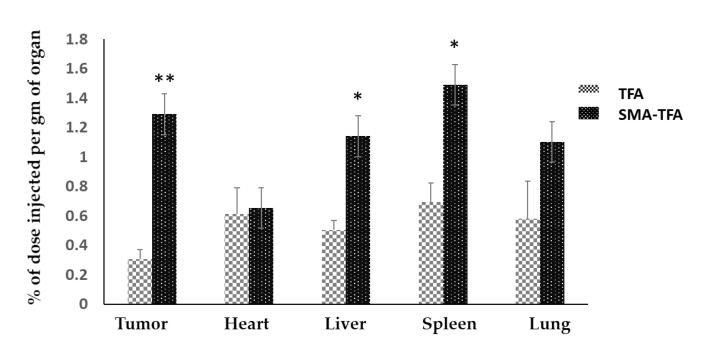
Tissue distribution of TFA and SMA–TFA at 24 h after intravenous injection of TFA or SMA–TFA (20 mg/kg) to Balb/c mice bearing 4T1 tumors (*n* = 5). Representation of the content of TFA per tissue expressed as the percentage of injected dose. * *p* < 0.05 and ** *p* < 0.01 when comparing TFA to SMA–TFA. Data are expressed as the mean ± SEM (*n* = 5).

To study corresponding organ distribution, TFA and SMA–TFA content was measured in tissues 24 h after injection (Figure 5). This time point reflects very low concentrations of both free and formulated TFA in the circulation, which can then serve as an appropriate time point to reflect the organ loads of both compounds after the dynamics of exchange between the blood compartment and various tissues. As shown in Figure 5, TFA was distributed in the heart, liver, spleen, and lungs, and tumors. As it can be observed, there was a noteworthy concentration of TFA following injection of SMA–TFA in the tumor, spleen, and lungs when compared to the free TFA injection, considering the percentage of the dose injected per organ (Figure 5). No significant statistical difference was observed in the heart (Figure 5). The increased amount of TFA observed in the liver and spleen following SMA–TFA injection may be due to high blood perfusion of the organs. In addition, the large vascular endothelial gaps present in the spleen favored the micelles’ accumulation [26]. The higher concentration of SMA–TFA inside the tumor tissue attributable to the EPR effect may enhance the therapeutic efficacy and promote a reduction in tumor growth.

## 3. Discussion

TNBC is a multifaceted subfamily of breast cancers possessing a very hostile clinical profile and exhibiting rapid proliferation, resistance to chemotherapeutics, high relapse risk, aggressive progression, a high rate of metastasis, and poor clinical outcome [3]. The lack of suitable biomarkers for personalized treatment (e.g., hormonal receptors) makes it difficult to treat. As a consequence, in spite of heterogeneity, TNBC has been homogenously treated mainly with chemotherapy. The mainstay of treatment for TNBC includes the consecutive use of an anthracycline/alkylator agent and subsequently a taxane (e.g., placlitaxel) or concomitant administration of an anthracycline (e.g., doxorubicin), an alkylator, and a taxane. Nanomedicine formulations include, among others, Doxil^®^ and Myocet^®^ (liposomal doxorubicin) and Abraxane^®^ (albumin nanoparticle of paclitaxel), indicated to treat advanced-stage breast cancer [27,28]. However, around 30% of patients have rapid relapse within three years following the first treatment [29]. Therefore, new approaches to overcome chemoresistance are needed. 

Duocarmycins are among the most potent anticancer agents known to date due to their potency, exceptional mechanism of action, and efficacy in multidrug-resistant tumor model; however, their translation to the market has been impaired due to severe side effects [16]. TFA is a *seco*-prodrug belonging to the duocarmycin family that dehydrochlorinates inside the cell with known DNA-alkylating and/or -intercalating potential. TFA displays strong cytotoxic activity against selected in vitro models of solid tumors and, at the same time, only mild toxicity against murine bone marrow cells. 

Because TFA potential in the management of TNBC has been poorly explored, the aim of the present paper was to investigate its effect in this subtype of breast cancer. To this extent, we set up an optimized synthesis of TFA suitable for obtaining gram quantities; in addition, TFA was loaded into SMA micelles to improve its water solubility, prevent the drug from enzymatic degradation, prolong its chemotherapeutic effect, and reduce the onset of drug resistance. 

Accordingly, a new deprotection method for the *O-*benzyl-protecting group of TFA was successfully set up (Scheme 1). In the presence of borontrichloride and of petamethylbenzene in DCM at −40 °C, the *O-*benzyl-protecting group was successfully removed to afford the desired TFA at 91% yield after chromatographic purification. The obtained TFA was then loaded on SMA micelles. Effective encapsulation of TFA was demonstrated by a loading capacity of 20% and a recovery of 65%. Molecular size (182 nm) was within the ideal 20–200 nm range requested to favor tumor accumulation via the EPR effect. The size distribution and loading were comparable to that obtained formerly by our research group [30,31]. Moreover, the particle size of the obtained SMA–TFA micelles was in the optimal range to sustain a long circulation time and extend their plasma half-life by preventing rapid elimination from the kidney. 

The surface charge of the obtained prepared SMA–TFA micelles was negative. Negative charge is considered to be biocompatible compared to positively charged nanoparticles that can interact with negative biomolecules in the plasma as well as the negative endothelial plasma membrane. The micellar formulation showed an initial release of around 20% of TFA attributable to drug association by means of polar interactions to the external shell surface of SMA followed by a sustained, slow-release rate of TFA for 72 h (Figure 2b). The SMA–TFA release curve demonstrates that our formulation showed an initial release of the drug enabling the rapid establishment of cytotoxic effects in the first two days of treatment (Figure 4a). Afterwards, the release becomes slow and steady, and the preparation thus can be considered as a reservoir for delivering a constant amount of TFA able to concentrate extracellularly at tumor tissues and hence prolong the exposure of tumor cells to effective doses of the drug.

In vitro studies confirm, for both free TFA and SMA–TFA, an outstanding cytotoxic activity towards the four breast cancer cell lines investigated. MDA-MB-468 was the least sensitive to both preparations, with an IC_50_ of 18.3 and 20.2 µM, respectively. Treatment of MDA-MB-231 and 4T1 with free TFA showed an IC_50_ of 4.6 and 5.9 µM, respectively, while SMA–TFA treatment induced slightly lower cytotoxicity (IC_50_ = 9.2 and 17.7 µM, respectively). Finally, the MCF7 cell line showed the highest sensitivity to both TFA and SMA–TFA with an IC_50_ of 4.55 and 4.31 µM, respectively. Interestingly, the four cell lines were extremely sensitive to both TFA and SMA–TFA treatments, showing cytotoxic activity in the low nanomolar range. 

Figure 4a clearly shows that treatment with SMA–TFA significantly inhibited the tumor growth in vivo, with almost no increase in the tumor size over the treatment period. The higher release at the beginning of the treatment (Figure 2b) might account for the rapid onset of SMA–TFA efficacy in the in vivo experiments, where a reduction of tumor size is observed within the first 2 days with a slight reduction in body weight (Figure 4b). Free TFA in the same in vivo model showed a significantly slowed growth when compared to control, which was far less effective than SMA–TFA treatment. Free TFA resulted in no significant weight loss in treated animals, indicating that it is relatively safe to use (Figure 4b). SMA–TFA, despite showing an initial mild weight loss within the first two days, showed no overall weight loss upon the duration of the treatment, suggesting also this preparation is safe in this animal model (Figure 4b).

In vivo biodistribution upon single intravenous administration demonstrated that TFA was broadly distributed in mice organs with peaks measured in highly vascularized organs such as the heart, spleen, and lungs (Figure 5). The lowest concentration, among analyzed organs, was observed in tumor tissues. SMA–TFA showed increased accumulation in the liver, spleen, and lungs compared to the free drug. However, as expected, a significantly increased accumulation of SMA–TFA was observed in tumor tissues when compared to the free drug (around four times). This might be attributable to the size of SMA–TFA micelles that increase the molecular size of the drug and, in turn, enhance its accumulation at the tumor site by the EPR effect [32]. Interestingly, both preparations showed comparable concentration in the heart. Given that cardiac tissues are expected to be the dose-limiting toxicity by inference to other molecules of the same family, the current nonmicellar preparation proved advantageous. Comparing the tumor/cardiac ratio between the free and micellar preparation showed that the SMA preparation is expected to be about four folds safer on myocardial tosses, as inferred by the 1.86 to 0.49 tumor/cardiac ratio in the preparation versus the free drug, respectively.

In conclusion, we reported an optimized method for the scale-up synthesis of TFA and an efficient way for its encapsulation in a micellar system. In vitro and in vivo studies suggest that both TFA and SMA–TFA possess high anticancer effects in TNBC models worthy of further investigation. Finally, the encapsulation of TFA offered a preferential avenue to tumor accumulation, increasing its concentration at the tumor tissues by around four times in comparison with the free drug by means of the EPR effect. Given that TNBC treatment still represents a major challenge and given the high frequency with which the tumor develops chemoresistance, findings reported in the present work may provide a new weapon against this often-fatal disease.

## 4. Materials and Methods

Polystyrene co-maleic anhydride (molecular weight = ~1600), *N*-(3-dimethylaminopropyl)-*N*-ethylcarbodiimide hydrochloride (EDAC), Hank’s balanced salt solution, Roswell Park Memorial Institute (RPMI) 1640 medium, fetal bovine serum (FBS), bovine serum albumin (BSA), and TrypLE express were bought from ThermoFisher Scientific (Dubai, UAE). L-glutamine and an antibiotic solution of penicillin/streptomycin were purchased from (Merck Hertfordshire, UK). All consumable materials such as Petri dishes, conical tubes (15 mL and 50 mL), cell culture flasks (25 cm^2^ and 75 cm^2^), and dialysis tubing were purchased from (Merck Hertfordshire, 120 Moorgate London, UK).

### 4.1. Chemistry

*O*-Benzyl–TFA was synthesized applying the optimized synthetic procedures previously reported by our group [33]. A solution of *O*-benzyl–TFA (2.0 g, 3.66 mmol) and pentamethylbeneze (1.62 g, 10.98 mmol, 3 eq) in anhydrous dichloromethane (50 mL) was cooled in a dry ice/acetone bath to −40 °C. Boron trichloride (1 M solution) in dichloromethane (7.3 mL, 7.3 mmol, 2 eq) was added dropwise to the solution in the ice bath over 5 min while stirring. Stirring was maintained at 40 °C for 45 min, then the reaction was quenched by the addition of 20.0 mL of a chloroform/methanol (10:1) mixture. The reaction mixture was allowed to warm to room temperature and was then concentrated under reduced pressure. The residue containing the crude product was purified by silica gel column chromatography (EtOAc/hexanes, 1:3) to yield pure TFA (1.52 g, 91%) as a buff powder. ^1^H-NMR (400 MHz, DMSO-*d*_6_): δ 3.79 (s, 3H, OCH_3_), 3.81 (s, 3H, OCH_3_), 3.93-3.98 (m, 4H, OCH_3_, H7), 4.07 (dd, *J* = 11.0, 3.4 Hz, 1H, H7′), 4.16 (ddt, *J* = 12.9, 8.6, 3.9 Hz, 1H, H8), 4.37 (dd, *J* = 11.0, 4.9 Hz, 1H, CH-Cl), 4.64-4.76 (m, 1H, CH’-Cl), 6.94 (d, *J* = 2.2 Hz, 1H, H3), 6.96 (s, 1H, H5), 7.01 (d, *J* = 2.2 Hz, 1H, H3′), 7.66 (s, 1H, NH), 7.81 (d, *J* = 2.2 Hz, 1H, H4′), 10.13 (s, 1H, H3′), 11.40 (d, *J* = 2.2 Hz, 1H, H2); ^13^C-NMR (101 MHz, DMSO-*d*_6_): δ 40,67 (C8), 47.10 (C7), 55.39 (CH_2_Cl), 56.42 (OCH^3^), 61.41 (OCH_3_), 61.58 (OCH_3_), 98.50 (C4′), 99.45 (C5), 105.17 (C3), 105.26 (q C), 106.36 (C3′), 113.58 (q C), 123.63 (q C), 125.72 (q C), 131.58 (q C), 139.52 (q C), 140.26 (q C), 142.98 (q C), 144.24 (C2), 149.61 (q C), 151.54 (q C), 151.78 (q C), 160.46 (CO).

### 4.2. SMA–TFA Micelles Synthesis

SMA micelles were synthesized using a well-established protocol with slight rearrangements [30]. Briefly, SMA powder was suspended in 1 M NaOH to reach a concentration of 10 mg/mL, heated at 70 °C for 3 h or until it became clear upon completion of anhydride hydrolysis. The solution of hydrolyzed SMA, cooled to room temperature, was then adjusted to pH 5.0, and EDAC in a 1:1 weight ratio with SMA was dissolved in distilled water (DW). TFA was dissolved in dimethyl sulfoxide (DMSO) at a 25% weight ratio to SMA. EDAC was added dropwise to the SMA solution simultaneously with TFA until a stable pH at 5.0 pH was achieved. The pH was raised to reach 11.0 and maintained until it became stable. The pH was then lowered to 7.4, and the clear solution was filtered 4 times using a Millipore Labscale TFF system equipped with a Pellicon XL 10 KDa cutoff membrane (Merck, Darmstadt, Germany) to a final volume of 50 mL. The freshly obtained SMA–TFA micelles were frozen at –80 °C and, after 24 h, lyophilized (5 mTorr and –55 °C) to afford a stable SMA–TFA powder.

### 4.3. SMA–TFA Micelles Characterization

#### 4.3.1. Loading of SMA–TFA

A standard calibration curve of TFA was prepared in DMSO in a range of concentrations from 1 to 25 µg/mL. Measures were performed at 322 nm. Drug content of SMA–TFA was determined by solubilizing SMA–TFA (1 mg/mL) in DMSO and measuring the absorbance at 322 nm in comparison with the standard curve. The loading was expressed as weight % of TFA in the final micelle compared to the total weight of recovered SMA–TFA. The SMA–TFA loading was determined as 20%. All the spectroscopic characterizations were carried out at room temperature (rt ≅ 25 °C) using a UV-2700 spectrophotometer (Shimadzu Corporation, Tokyo 101-8448, Japan).

#### 4.3.2. Size and Charge of SMA–TFA Micelles

SMA–TFA micelles (1.5 mg/mL) were solubilized either in NaHCO_3_ (0.1 M, pH 7.8) to determine the size or distilled water to estimate the charge. The width of distribution (polydispersity index, PDI), together with the zeta potential of SMA–TFA, was measured by a Malvern ZEN3600 (Malvern Instruments Inc., Westborough, MA, USA). Measurements from three independent experiments were conducted in triplicate and at room temperature (rt ≅ 25 °C).

#### 4.3.3. Release of TFA from SMA–TFA Micelles 

The release rate of free drug (TFA) from the SMA–TFA micellar system was measured in PBS. A 2 mg amount of the prepared micelles was dissolved in 2 mL of PBS and inserted into a 10 kDa cutoff dialysis membrane that was submerged in 20 mL of PBS for 72 h. At specified time points, the surrounding water was collected from outside the dialysis bag and replaced with PBS, and the absorbance was measured at 322 nm.

### 4.4. Cell Culture

MDA-MB-231, MDA-MB-468, 4T1, and MCF7 cell lines were purchased from American Type Culture Collection (ATCC) (Manassas, VA, USA). RPMI medium supplemented with 5% fetal bovine serum (FBS) was used to culture the cell lines while being maintained in a humidified atmosphere at 37 °C, 5% CO_2_.

### 4.5. Antiproliferative Effect of Free TFA and SMA–TFA Micellar Formulation in Breast Cancer Cells

Cells were seeded in 96-well plates (density: 4T1, 5 × 10^3^; MDA-MB-231, 5 × 10^3^; MDA-MB-468, 5 × 10^3^; MCF7, 5 × 10^3^ cells/well) and incubated for 24 h at 37 °C in 5% CO_2_ and subsequently treated with different of concentrations of TFA (10^−7^ to 10^−10^ M) or SMA–TFA (10^−7^ to 10^−10^ M). Cytotoxicity was assessed after 48 h incubation using sulforhodamine B (SRB) assay as described previously [25]. Cells were fixed using 10% trichloroacetic acid and stained with SRB. The cytotoxicity experiments were performed in triplicate (*n* = 3). The 50% growth inhibition (IC_50_) was then assessed by using SRB assay after 48 h incubation. Data are represented as the mean ± SD of three independent experiments of each cell line.

### 4.6. In Vivo Antitumor Effects of TFA and SMA–TFA 

Female Balb/c mice (6–12 weeks old, mean weight = 20–25 g) were supplied by the Laboratory Animal Care Facility of the Arabian Gulf University (AGU), Bahrain. All animals were kept under standard conditions, including controlled temperature (25 °C) and a 12 h light/dark cycle, and had free access to food and drinking water ad libitum. All animal experiments were performed based on the rules and regulations of the Arabian Gulf University Animal Care Policy and approved by the Research and Ethics Committee, REC Approval No: G-E001-PI-12/16.

To propagate the tumor, female Balb/c mice (*n* = 3) were injected with one million 4T1 mammary carcinoma cells bilaterally (right and left side) on the flanks. The tumor was then collected and cut down into small pieces with an average size of 1–3 mm^3^ in sterile PBS to sustain tumor viability. Following this, 5 mice of each group were shaved, anesthetized, and inoculated with one small piece of the 4T1 tumor tissue subcutaneously. When the tumors reached 100 mm^3^ in size, mice were randomly distributed into three groups (*n* = 5 in each group (negative control, TFA, and SMA–TFA)) and subjected to drug treatment. TFA was administered at a dose of 3 mg/kg via the tail vein, while SMA–TFA dissolved in PBS at a dose of 3 mg/kg (TFA equivalent dose) was given by IV injection. The first day of drug administration was set as Day 0. Tumor volume was measured by a manual caliber, and the volume was estimated by using the formula:

V (mm^3^) = ((transverse section (W)^2^ × longitudinal cross section (L))/2)

Tumor volumes were normalized by using the initial tumor volume and represented as the mean ± standard error of the mean (SEM). Additionally, the body weight of mice was measured every day and normalized daily for 9 days.

### 4.7. In Vivo Biodistribution of TFA and SMA–TFA Micelles

Female Balb/c mice were implanted with 4T1 cell tumors (1–3 mm^3^) tumor size, bilaterally on the flanks. When tumors reached 100 mm^3^, mice were randomly distributed into two groups (5 animals per group). The animals were injected with either TFA or its equivalence in SMA–TFA at 20 mg/kg via the tail vein. Mice were euthanized 24 h after the treatment, and organs were collected. Internal organs (heart, lungs, liver, and spleen) and tumor tissue were analyzed for TFA content. SMA–TFA was extracted using the methodology previously described [34]. Briefly, tissues were minced, weighed, and snap-frozen before being pulverized. Frozen tissue powder (1 mg) was resuspended in 67% ethanol and 4 M HCl (1 mL). The suspension was incubated at 70 °C for 0.5 h, sonicated, and centrifuged to extract TFA from tissue samples. TFA content was determined by absorbance at 322 nm and compared to a TFA calibration curve. TFA content was normalized to the weight of tissue and to the total weight of the organs from which it was extracted.

## Figures and Tables

**Table 1:** Characterization of SMA–TFA ^1^.

Micelle	Recovery	Loading (wt/wt)	Size (nm)	PDI ^2^	Zeta Potential (mV)
SMA–TFA	65%	20%	181.7 ± 80.32	0.163	–57.7

^1^ Data are shown as the mean ± standard deviation (SD). Values are the mean of triplicate experiments; ^2^ PDI = polydispersity index.

**Table 2:** Experimental IC_50_ values (nM) of free TFA and SMA–TFA towards human MDA-MB-231, MDA-MB-468, 4T1, and MCF-7 cells.

Cells	TFA IC_50_ (nM)	SMA–TFA IC_50_ (nM)
MDA-MB-231	4.6	9.2
MDA-MB-468	18.3	20.2
4T1	5.9	17.17
MCF7	4.55	4.31

IC_50_ value determination was performed using GraphPad Prism. Data are reported as IC_50_ values in nM.

## Supplementary Materials

The following are available online and contain ^1^H-NMR and ^13^C spectra of tafuramycin A.

## Sample Availability

Samples of TFA and SMA–TFA are available from the authors.
