# Supplementary material for: Selective Targeting of Breast Cancer by Tafuramycin A Using SMA-Nanoassemblies
Source: Molecules. 2021 Jun 9;26(12):3532. doi: 10.3390/molecules26123532 (PMC8227162; doi:10.3390/molecules26123532)

Supporting Information

Selective Targeting of Breast Cancer by Tafuramycin A Using SMA-Nanoassemblies

Ibrahim M. El-Deeb [1], Valeria Pittala [2,*], Diab Eltayeb [3] and Khaled Greish [3,*]

[1] Department of Medical Sciences, Royal College of Surgeons in Ireland, Medical University of Bahrain, Busaiteen 228, Bahrain; imeldeeb@gmail.com
[2] Department of Drug and Health Science, University of Catania, Catania 95125, Italy
[3] Department of Molecular Medicine, Arabian Gulf University, Manama 329, Bahrain; khaledfg@agu.edu.bh
* Correspondence: valeria.pittala@unict.it (V.P.); khaledfg@agu.edu.bh (K.G.); Tel.: +39-095-7384269 (V.P.); Tel.: +973-17-237-393 (K.G.)

Content:

1. ^{1}H NMR spectrum of Tafuramycin A……………………………………………………………………….2
2. ^{13}C NMR spectrum of Tafuramycin A………………………………………………………………………3

^{1}H NMR spectrum of Tafuramycin A

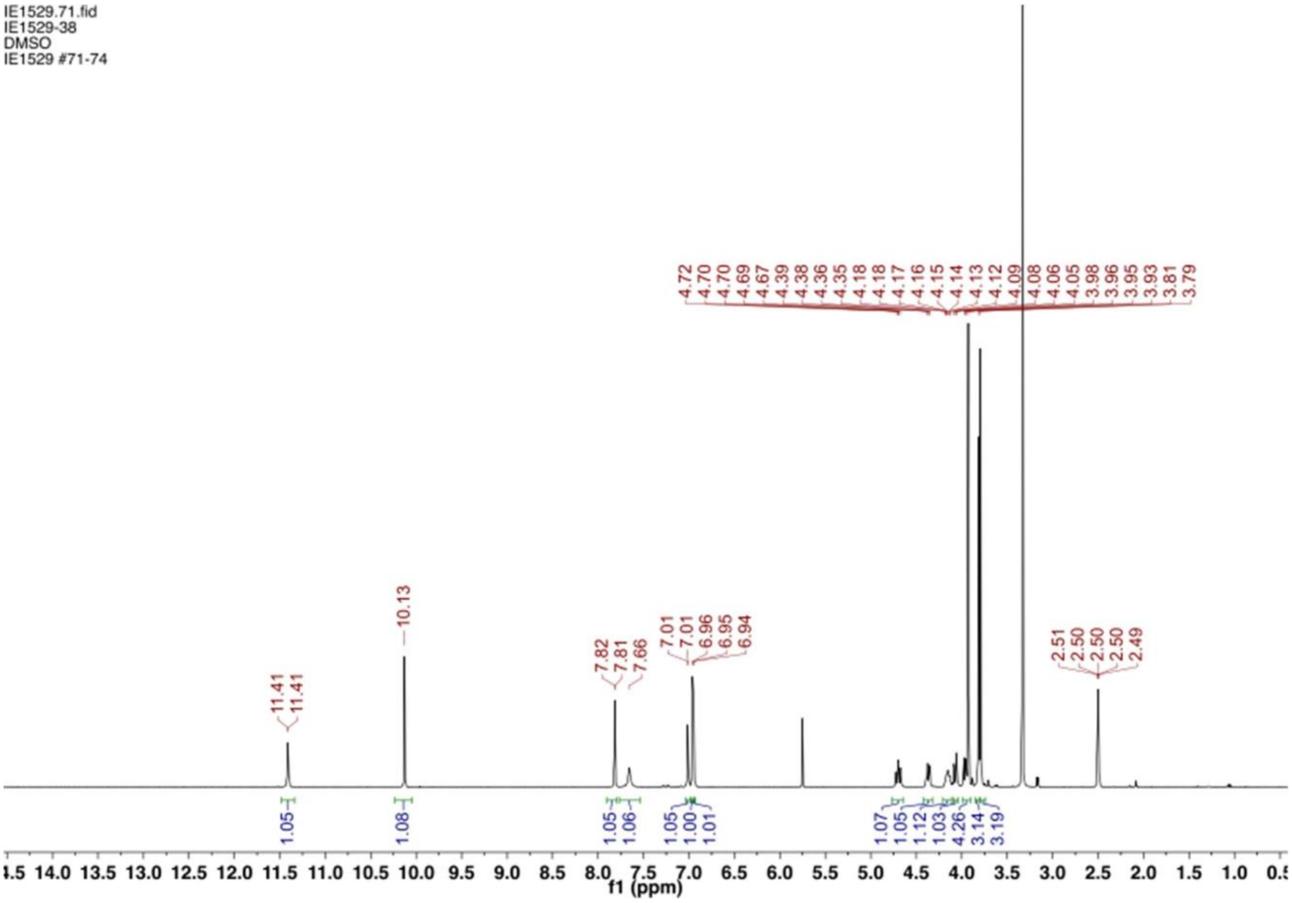

^{13}C NMR spectrum of Tafuramycin A

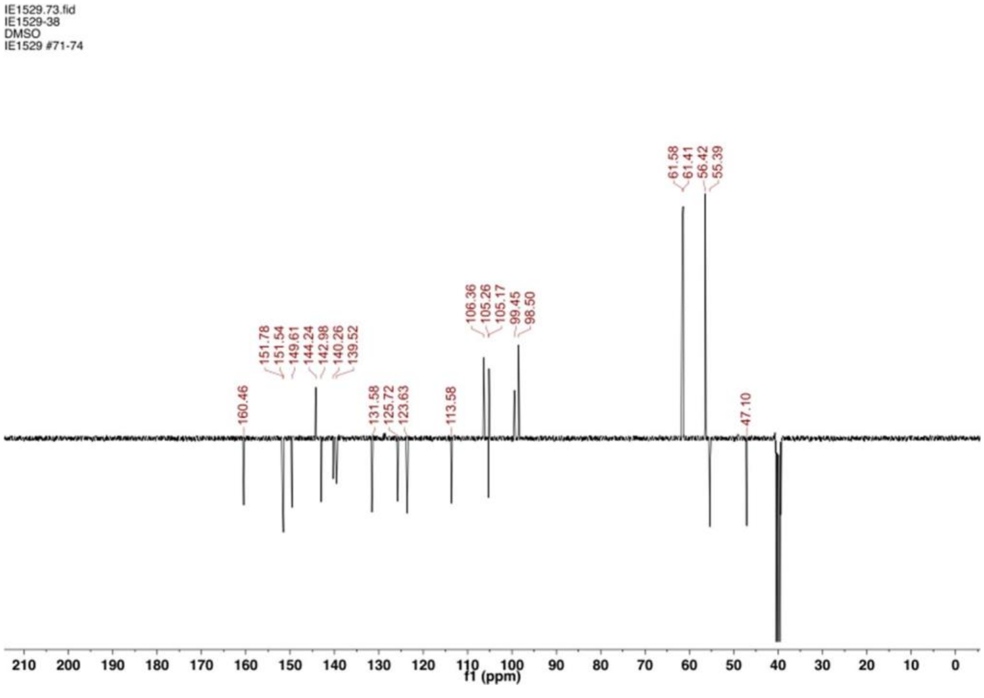

Supplement: Supplementary file 1 [file molecules-26-03532-s001.zip › molecules-1238409-supplementary-1.pdf]
